# Three-Dimensionally Printed Microsystems to Facilitate Flow-Based Study of Cells from Neurovascular Barriers of the Retina

**DOI:** 10.3390/mi15091103

**Published:** 2024-08-30

**Authors:** Adam Leverant, Larissa Oprysk, Alexandra Dabrowski, Kelly Kyker-Snowman, Maribel Vazquez

**Affiliations:** Department of Biomedical Engineering, Rutgers, The State University of New Jersey, Piscataway, NJ 08854, USAlmd346@scarletmail.rutgers.edu (L.O.);

**Keywords:** rapid prototyping, endothelial cells, retinal neural cells, morphology, survival

## Abstract

Rapid prototyping has produced accessible manufacturing methods that offer faster and more cost-effective ways to develop microscale systems for cellular testing. Commercial 3D printers are now increasingly adapted for soft lithography, where elastomers are used in tandem with 3D-printed substrates to produce in vitro cell assays. Newfound abilities to prototype cellular systems have begun to expand fundamental bioengineering research in the visual system to complement tissue engineering studies reliant upon complex microtechnology. This project used 3D printing to develop elastomeric devices that examined the responses of retinal cells to flow. Our experiments fabricated molds for elastomers using metal milling, resin stereolithography, and fused deposition modeling via plastic 3D printing. The systems were connected to flow pumps to simulate different flow conditions and examined phenotypic responses of endothelial and neural cells significant to neurovascular barriers of the retina. The results indicated that microdevices produced using 3D-printed methods demonstrated differences in cell survival and morphology in response to external flow that are significant to barrier tissue function. Modern 3D printing technology shows great potential for the rapid production and testing of retinal cell responses that will contribute to both our understanding of fundamental cell response and the development of new therapies. Future studies will incorporate varied flow stimuli as well as different extracellular matrices and expanded subsets of retinal cells.

## 1. Introduction

Contemporary microtechnology has transformed the nature of quantitative study in many physical and life science disciplines (reviewed in [1,2,3]). Microdevices offer precise control of extracellular environments and manipulation of sub-microliter volumes to examine fundamental biological processes. A merger of microscale systems with tissue engineering has produced sophisticated microfluidics to study critical cellular behaviors, including adhesion and cohesion [3,4], cell–cell connectivity and communication [5,6], and migratory responses to a variety of externally applied fields (reviewed in [7,8,9]). Fabrication techniques have similarly evolved in tandem with complex biological application to herald more integrative technologies, such as organ-on-a-chip devices and micro-physiological systems that recapitulate key physiological features of tissues (reviewed in [10,11]). In a complementary direction, a growing community has begun to produce microscale tools independent of specialized facilities and machinery [12,13,14]. These include paper microfluidics [15,16], traditional machining [17,18], stereolithography [19,20], and other rapid prototyping [21,22] to accommodate live cells.

Additive manufacturing has become ubiquitous through commercial and cost-effective 3D printers that rapidly fabricate microscale devices using a variety of resins, polymers, slurries, and biomaterials [23,24,25]. The accessibility of 3D printers in public and private maker spaces has revolutionized rapid prototyping to produce a wide range of functional and practical engineering products, as well as personal items, art, toys, and more [26,27,28]. Moreover, the systems have made scientific inquiry truly accessible to communities with low resources [29,30,31] as well as expanded microtechnology into a wider range of applications in biomedical engineering and regenerative medicine [6,32,33]. Today, 3D-printed microsystems have been adapted for in vitro study using whole organisms, tissues, and cells through soft lithography, i.e., a collection of techniques that enable the fabrication of structures using elastomers [34]. Recent projects have examined the performance of microfluidic devices manufactured from polymers (most commonly poly-dimethyl siloxane, PDMS) using 3D-printed molds rather than conventional, mask-enabled silicon wafers [35,36,37,38]. Recent systems have been applied to examine cell behaviors, as 3D-printing methods enable the rapid development of in vitro systems to visualize responses. Rapidly prototyped projects have facilitated the development of cell-laden hydrogels [39,40], synthetic environments to study cellular movement [41], and cell–scaffold colonization [42], as well as generated microfluidic componentry to facilitate cell culture and cell visualization [43,44].

With rising bioengineering interest in regenerative medicine [45], the visual system has become increasingly studied using microtechnology [46,47]. However, few laboratories have adapted rapid prototyping to study cell behaviors of critical tissue, such as the retina responsible for the phototransduction of light into vision. As shown in Table 1, small numbers of groups have used traditional photolithography to examine the migration of retinal progenitors [48,49,50,51] and incorporate nanotechnology to examine communication across retinal cells [52]. Newer studies have used 3D printing to examine hydrogel deposition and fibrous remodeling in retinal-like structures [53,54] but few have used additive manufacturing to aid in the development of therapies [55].

Rapid prototyping is exceptionally well suited for models of the visual system at the micro- and mesoscale, i.e., with characteristic lengths between 50 μm and 500 μm [35,67,68]. This includes critical barrier tissue such as the inner blood–retinal barrier (BRB), a neurovascular tissue that supplies the retina with oxygen and nutrients from circulating blood to meet its high metabolic demands for vision. The BRB has been examined using a variety of complex, microfabricated systems and diverse subsets of constituent cells (reviewed in [69]). However, a fundamental aspect that remains understudied is the effect of physiological flow rates on the behaviors of different BRB cell types. Hydrodynamics become particularly relevant in disorders fueled by chronic hypertension and hyperglycemia, both associated with rising ocular disorders of glaucoma and diabetic retinopathy [70,71].

**Figure 1:**
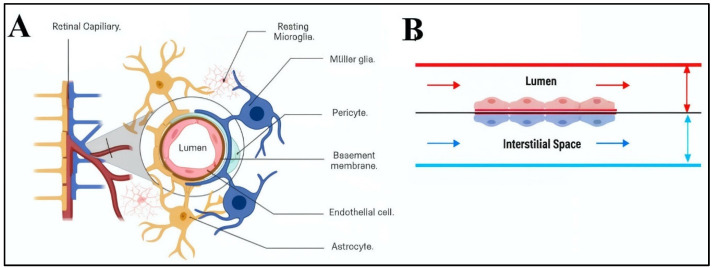
Schematic of key cellular and structural features of the inner blood–retinal barrier (BRB). (**A**) This neurovascular barrier tissue is primarily composed of endothelial cells and pericytes within retinal capillaries, as well as astrocytes and Muller glia that reside within neural tissue. (**B**) Side view of circulating blood flow that exerts continuous shear stress upon endothelial cells that line the lumen (**top**). Also shown is a side view of cognate neuroglia exposed to shear stress from interstitial flow of neural tissue (**bottom**).

The vascular BRB is composed of endothelial cells that line the inner surfaces of retinal capillaries and pericytes that cooperatively regulate angiogenic responses [72], as shown in Figure 1. Neuroglia on the retinal side include astrocytes that line the nerve fiber layer and Muller glia that reside within the retina [8,73]. These neuroglia work collaboratively to maintain the BRB’s integrity by stabilizing tight and gap junctions between cells and regulating metabolism and retinal homeostasis [74]. Capillary and interstitial flow rates are well known to vary by an order of magnitude in vivo and can have dramatic effects on constituent cells [75,76]. Rapid prototyping enables the development of micro- and mesoscale systems able to connect with flow pumps to vary volume flow rates, pressures, and examine the behaviors of BRB cells in physiological and pathological conditions.

This project developed microfluidic systems to examine the effects of flow on BRB cell types using PDMS soft lithography and 3D-printed molds made from resin (polylactic acid, PLA) and plastic (polyethylene terephthalate, PETG). The systems were compared to the glial line (gLL) system previously described by our group and fabricated via metal machining as well as traditional photolithography [55,77]. Our experiments examined the average surface roughness of the channel interstitial spaces, with and without a laminin coating, the viability of retinal endothelial cells (RECs) and retinal neural cells (RNCs) within each 3D-printed device, and morphological changes in response to imposed shear stress. The results demonstrate comparable cell viability across devices as well as measurable changes in cell morphology. Taken together, our results highlight the underexplored impact of 3D-printed devices to the bioengineering study of retinal cell responses using imposed flow. The insights gained from these microdevices will increase contributions from diverse bioengineering groups to spur the development of much-needed therapies for retinal disease.

## 2. Materials and Methods

### 2.1. Established Glial Line (gLL) Design

**Figure 2:**
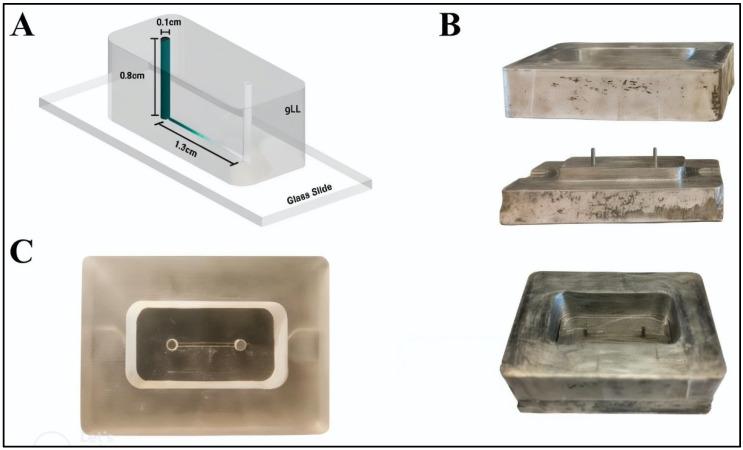
Summary of the glial line system, known as the gLL. (**A**) Schematic of the gLL used as 3D model for manufacturing. A PDMS elastomer is bonded to a glass microscope slide to produce a closed microchannel in between two volumetric reservoirs, as previously described by our group. (**B**) The mold used to cure the elastomers needed to produce gLL devices was fabricated using metal milling via computer numerical control (CNC) in the three parts shown. (**C**) A top view of the final gLL system produced by curing PDMS within the metal molds.

This project used an existing design from our laboratory, the glial line (gLL) system [55], to represent the anatomical scale of microenvironments in which adult retinal cells reside, i.e., approximately 200 µm in characteristic length. Tests used the system design to examine performance of PMDS-cast microsystems produced using molds fabricated from different methods. In brief, the gLL is composed of two cylindrical reservoirs, each 1 mm in diameter, 0.8 cm in height, and 6.0 µL in volume. The reservoirs are connected by a 1.3-mm-long microchannel that is 180.75 ± 4.7 μm in height and 207.3 ± 6.6 μm, for an equivalent hydraulic diameter of 193.1 µm. The original gLL was fabricated using a milled, aluminum mold via computer numerical control (CNC), followed by elastomeric molding with polydimethylsiloxane (PDMS), as shown in Figure 2. A 3-axis TRAK DPM SX2P Bed Mill with the ProtoTRAK SMX CNC was used to mill the gLL design on aluminum, with a tolerance range of ±40 µm, as described.

**Figure 3:**
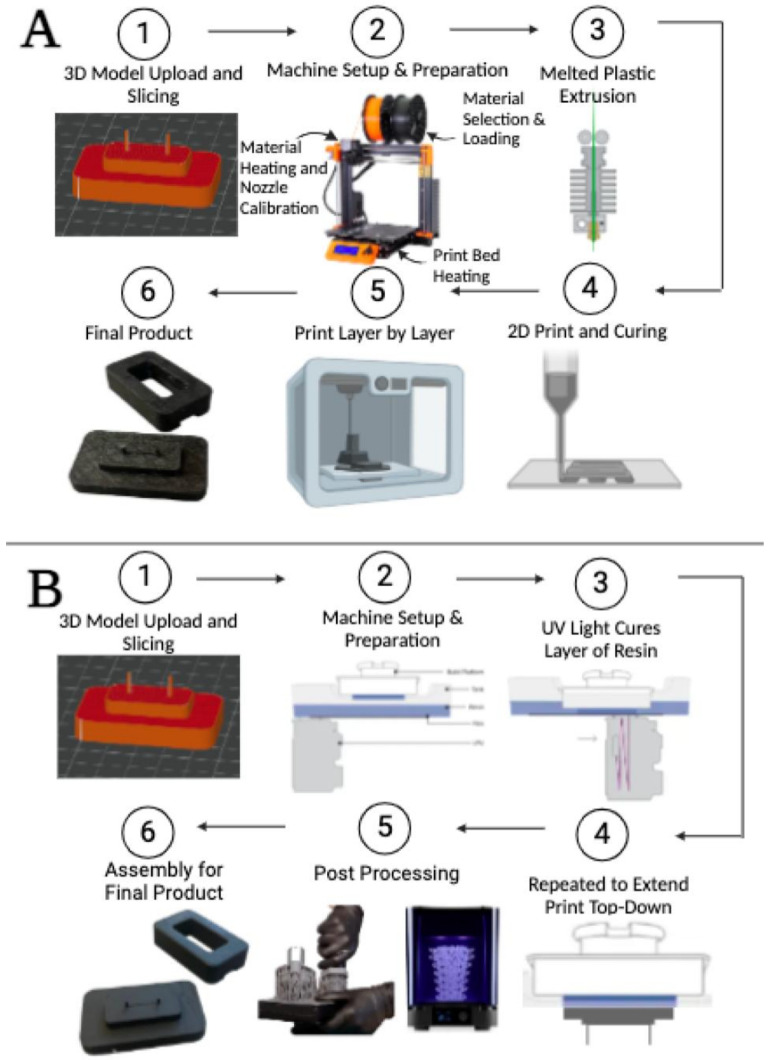
Summary of key steps in the rapid prototyping of 3D molds used for elastomeric soft lithography. (**A**) Fused deposition modeling (FDM) was used to manufacture plastic molds of polyethylene terephthalate glycol (PETG), wherein the 3D device model was converted to gcode for melted plastic extrusion and printed layer by layer. (**B**) Stereolithography (SLA) was used to develop resin molds of polylactic acid (PLA) via digital upload of the 3D device model, UV curing of layered resin, and two rounds of post processing in isopropyl alcohol.

For the three-dimensionally printed plastic mold, the gLL design mold was printed using polyethylene terephthalate glycol (PETG), a thermoplastic polyester commonly used in commercial printers for its significant resistance to heat and solvents, as well as durability and formability [78]. Molds were manufactured by fused deposition modeling (FDM) using a Bambu X1 Carbon 3D printer with a 0.4 mm hardened steel nozzle and Engineering Plate printing surface with a layer height of 0.16 mm, monotonic linear surface pattern, and grid pattern sparse infill at 15% density. Three-dimensional modeling files were converted to gcode with the Bambu Studio slicer with the above settings, then printed, as shown in Figure 3A.

For the three-dimensionally printed resin mold, the gLL design mold was also printed using stereolithography (SLA), or vat photopolymerization, where a light source was used to cure liquid resin into hardened plastic. Molds were manufactured using a Formlabs Form 2 Resin Printer for vat photopolymerization of conventional polylactic acid (PLA) resin [79]. As shown in Figure 3B, 3D modeling files were converted to gcode with the PreForm slicer at 100 μm resolution, printed in clear resin, then washed twice by immersion in isopropyl alcohol.

### 2.2. Device Reagents

Devices were fabricated using elastomeric molding with commercial polydimethylsiloxane (PDMS; Cat. No. 1020992-312, VWR, Radnor, PA, USA). An elastomer base-to-curing agent ratio of 1:9 (weight per volume) was mixed and vacuum desiccated for 15 min to remove excess bubbles. After degassing, approximately 5-mL of the mixture was poured into a gLL mold and allowed to polymerize via oven (100 °C) for 15 min. Once polymerized, the elastomer was manually removed from the mold and exposed to corona plasma treatment for 5 s. The elastomer was then firmly pressed upon a microscope glass slide, which had previously been chemically sterilized and corona-treated for 5 s, to generate an ozone-bonded, closed system.

The inner surfaces of PDMS elastomers cast upon the different gLL molds were coated with laminin (Corning, Cat. No. 354232, Bedford, MA, USA) at a concentration of 15 µg/mL diluted in phosphate buffered saline (PBS). Laminin was chosen because it is a critical component of the basement membrane of the blood–retinal barrier and plays pivotal roles in the viability, adhesion, and communication of constituent retinal cells [62]. A 100-µL volume of this extracellular substrate was loaded into the devices via syringe and allowed to crosslink at 37 °C overnight in a 5% CO_2_ incubator. Excess laminin solutions within gLL interstitial spaces were then aspirated out and devices were cleaned via manual PBS wash using a 1-mL syringe. 

### 2.3. Modeling and Validation of Flow within Microdevices

**Figure 4:**
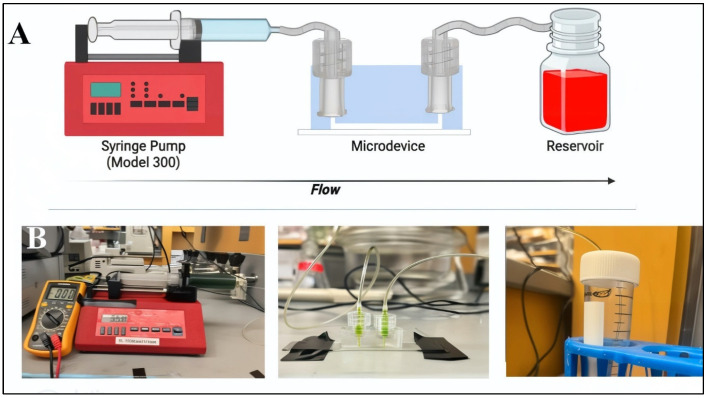
Graphical summary of the flow system used to examine behavior of retinal endothelial and retinal neural cells from the inner blood–retinal barrier. (**A**) Schematic of pump-driven flow applied within the microdevice and collection of cell media. (**B**) Images of experimental flow system and representative microdevice.

Flow was introduced through the source reservoir of the device to initiate flow along the microchannel towards the opposite reservoir. A 60-mL volume of cell media was loaded into the pumping device via syringe, as per Figure 4.

Flow within a previously sterilized syringe was connected to the gLL via Luer Locks, as shown. Different volume flow rates were used to pump media into the device reservoir and collected in an Eppendorf tube for further biological testing, as desired. Flow was maintained for six hours upon cell monolayers and promptly followed by cell staining and imaging. The induced flow was well-described by the Navier Stokes of Equation (1):

ρ(∂u/∂t + u∙∇u) = −∇P + μ∇^2 u + ρg(1)

where ρ is density, u is velocity, μ is viscosity, P is pressure, and g is gravity. The dimensions, fluid properties, and time scale of experiments facilitated approximation of one-dimensional, incompressible flow at steady state. Moreover, application of no-slip axisymmetric boundary conditions reduced the governing equation to the well-established Poiseuille Flow model [80], where the pressure gradient and shear stress are defined by Equations (2) and (3), respectively:

dP/dz = (8μQ/(πR^4))(2)

τ = (4μQ/(πR^3))(3)

where P is pressure, τ is shear stress, μ is viscosity, Q is applied volume flow rate (via pump), R is hydrodynamic radius of the microchannel, and z denotes the axial length.

The flow velocity within the channel cross section was experimentally validated by measuring the velocity of 10-μm-diameter beads (Thermo Fisher Scientific, Cat. No. F8842, Eugene, OR, USA) that were imaged via microscope camera every 60 s for the first 3 h and every 60 min thereafter. Flow was induced using a syringe pump (New Era Pump, NE-1600, New York, NY, USA). Measurement of bulk velocity used a sample size of n = 50 beads at three separate sections of the microchannel, per device and experimental conditions. Variance in velocity data was determined by the root mean square error between solutions of Poiseuille Flow at the different channel sections, as previously conducted by numerous groups, including our own (reviewed in [33]).

### 2.4. Cell Culture

Experiments used cultured rat capillary endothelial cells (RECs, CellBiologics, RA6065, Chicago, IL, USA) maintained in Complete Rat Endothelial Cell Medium (CellBiologics, Cat. No. M1266, Chicago, IL, USA). The media contained 0.5-mL of epidermal growth factor (EGF), 0.5-mL of vascular endothelial growth factor (VEGF), 5.0-mL of Antibiotic-Antimycotic Solution, and 10.0-mL of fetal bovine serum (FBS). RECs were incubated at 37 °C and 5% CO_2_ and cultured in T-75 flasks (VWR Cat No. 0062-868, Radnor, PA, USA). Cells at 80–90% confluency were dislodged and re-suspended in media. Cell solutions were then inserted into sterile microenvironments of PDMS devices at a density of 5 × 10^6^ cells per mL to form a near-confluent monolayer.

Retinal neural cells (RNCs) were represented by cultured r28 cells (Kerafast, Cat. No. ENW001, Boston, MA, USA), an immortalized cell line derived from a rat model and used extensively by our group and others in retinal study [6,81]. RNCs were maintained in Dulbecco’s Modified Eagle’s medium (DMEM) (Cat. No. 30-2002, ATCC, Manassas, VA, USA) containing 4 mM L-glutamine, 4500 mg/L glucose, 1 mM sodium pyruvate, and 1500 mg/L sodium bicarbonate. Cell media were supplemented with 10% FBS (Invitrogen-Gibco, Rockville, MD, USA) and incubated at 37 °C and 5% CO_2_. RNCs were cultured in T-75 flasks (VWR Cat No. 0062-868, Radnor, PA, USA) and passaged at 80–90% confluency into sterile microenvironments of PDMS devices at a density of 5 × 10^6^ cells per mL to form a near-confluent monolayer. 

### 2.5. Measurement of Cell Viability and Morphology

Viability of RECs and RNCs was assessed using a Live/Dead assay (Thermo Fisher Scientific, Cat. No. L3224, Carlsbad, CA, USA). The assay discriminated cells via staining with green-fluorescent calcein-AM to indicate intracellular esterase activity (live) and red-fluorescent ethidium homodimer-1 to indicate loss of plasma membrane integrity (dead). Cells were additionally stained with the nuclear DAPI stain (Thermo Fischer Scientific, Cat. No. 62248, Carlsbad, CA, USA) for better visualization.

Cell morphology was evaluated using the cell shape index (CSI), a dimensionless parameter widely used by our group and others [82] to quantify the roundness of a cell, as defined in Equation (4):

CSI = ((4πA_S)/P^2)(4)

where AS is the surface area and P is the perimeter of the cell. The value of the CSI ranges from 0 to 1, where values close to 1 represent a perfectly rounded cell and values approaching 0 denote a purely bipolar and elongated cell.

### 2.6. Fluorescence, Imaging, and Analysis

An inverted epifluorescence microscope (Leica DMi8) was used to observe cell behavior over time and to perform optical analyses with a cooled CCD camera (Leica Microsystems, DFC7000 GT, Chicago, IL, USA) via a 20× objective. Images were evaluated using ImageJ with 12-bit data.

### 2.7. Statistical Analysis

Differences among adherent cell groups were evaluated using one-way analysis of variance (ANOVA) and post hoc test (Tukey). A one-way ANOVA test at the 95% confidence interval assessed statistical significance across devices manufactured upon different molds and flow rates. Each data set was gathered from a representative total of n = 25 cells per device, using 5–7 independent devices per experimental mold and condition. Values are reported using mean and standard deviation. The post hoc Tukey tests were used to determine statistical significance between conditions, where *p*-values < 0.05 were denoted by an asterisk, *, and *p* < 0.01 were marked with a double asterisk, **.

## 3. Results

### 3.1. Elastomeric Devices Produced from 3D-Printed Molds Exhibited Greater Variance than Devices Produced from Original Metal Molds

Dimensions of the gLL system were selected to represent the anatomical scale of the extracellular environment in which adult retinal neural cells (RNCs) reside and interact with retinal endothelial cells (RECs) of the BRB tissue. As per Figure 1, the width of the microchannel approaches retinal thickness, through which RNCs span to establish connections with surrounding neuronal cells and interact with cognate RECs. The gLL system with these larger mesoscale features was reproduced via elastomeric molding upon milled aluminum molds, shown in Figure 2. PDMS curing upon the metal molds created microchannels that were rectangular in cross section, with an average length of 1.48 ± 0.05 cm, height of 239.1 ± 6.75 μm, and average width of 180.7 ± 4.6 μm. This rectangular cross section exhibited a height to width ratio of 1.32. These dimensions produced PDMS elastomers with an average hydraulic diameter of 205.7 ± 3.07 μm, as listed in Table 2. Average dimensions of the volumetric reservoirs connected to either end of the microchannel were 1.00 ± 0.05 μm in diameter and 4.10 ± 0.05 μm in height. By contrast, elastomeric channels produced from using gLL molds that were 3D printed using SLA with PLA resin were 1.21 ± 0.05 cm in length, 250.9 ± 32.8 μm in height, and 255.2 ± 8.1 μm in width. The microchannels exhibited a cross section that was more square-like with an average height-to-width ratio of 0.98. This produced an average hydraulic diameter of 255.2 ± 8.1 μm, which was ~24% larger than the PDMS elastomer produced by the original metal mold. The average dimensions of the volumetric reservoirs connected to either end of this microchannel were 1.67 ± 0.05 μm in diameter and 6.73 ± 0.05 μm in height, for a ~29% increase in volume. Lastly, elastomers produced using molds that were 3D printed using FDM and PTEG plastic resulted in channels that were 1.19 ± 0.05 cm in length, 398.9 ± 10.8 μm in height, and 207.8 ± 4.0 μm in width. These microchannels also displayed the largest cross section with an average height-to-width ratio of 1.92. In addition, the average hydraulic diameter of 273.3 ± 6.0 μm was larger than that produced using the SLA mold and 33% larger than the PDMS elastomer produced using the original metal mold. The average dimensions of the volumetric reservoirs connected to each channel end were 1.66 ± 0.05 μm in diameter and 6.21 ± 0.05 μm in height, for a ~21% increase in volume from the original.

### 3.2. Elastomers Produced Using 3D-Printed Molds Exhibited Wide Variance in the Average Roughness of Microchannel Inner Surfaces

**Figure 5:**
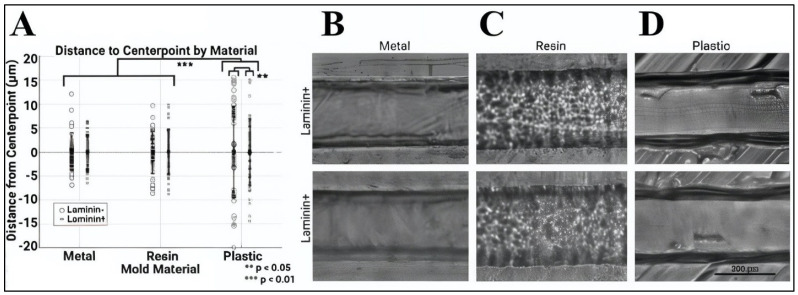
Surface roughness of inner channels of elastomeric microdevices manufactured using 3D molds made of metal, resin, and plastic. (**A**) Data illustrate measurements from the channel center as determined via optical microscopy for inner channel surfaces coated with (+) and without (−) laminin. Representative images of the microfluidic channel manufactured using molds of (**B**) metal, (**C**) resin, and (**D**) plastic, with (+) and without (−) laminin coating (Scale bar = 200 μm). An average of n = 25 measurements were gathered per device, using 5–7 devices per mold type and laminin coating condition. Statistical significance is denoted by *p* < 0.05 (**) and *p* < 0.01 (***).

PDMS devices produced via curing upon the 3D-printed molds were next measured for surface roughness of the channel’s inner surfaces. The devices were measured with and without a laminin coating to determine their utility for cell adhesion and survival. Figure 5A illustrates the distance measured from the surface of the channel center point to its respective mean height. As shown, PDMS devices produced from the metal molds exhibited an average surface roughness with a deviation of ±15 μm, while elastomeric channels produced from PLA resin molds and PTEG plastic molds exhibited a surface roughness with a deviation of ±19 μm and ±30 μm from average channel heights, respectively. The values of the surface roughness of elastomeric channels cured using SLA resin were insignificant from those of channels cured upon metal molds (*p* > 0.05). By contrast, the surface roughness of channels produced using PTEG plastic molds was significantly different from those produced using metal molds (*p* < 0.05). Moreover, only elastomers produced using plastic molds exhibited significant differences in surface roughness between non-coated and coated laminin surfaces (*p* < 0.01). As a result, the remainder of this study focused on elastomers produced from resin molds using PLA.

### 3.3. Bulk Flow within Elastomeric Devices Approached Analytical Flow Solution

**Figure 6:**
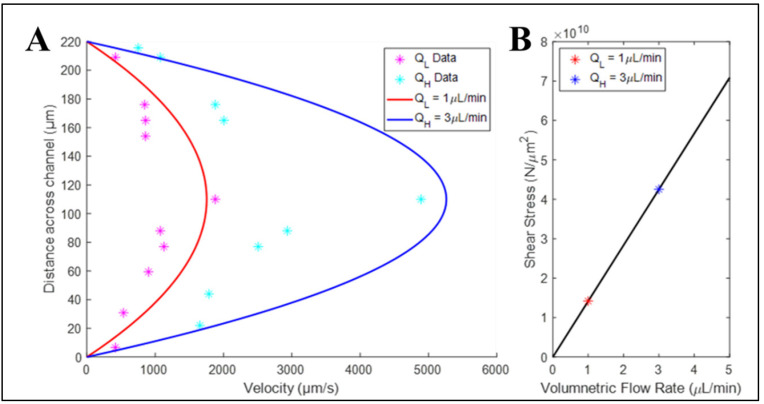
Measurement of particle flow within 3D-printed microsystems alongside analytical flow model. (**A**) Measured velocity of individual microbeads (*) plotted against one-dimensional analytical solutions to Poiseuille Flow with low (QL) and high (QH) volume flow rates. (**B**) Analytical solution to imposed shear stress and QL and QH in the same system.

Our tests next examined the differences in bulk flow within elastomeric devices fabricated using 3D-printed SLA resin molds. Figure 6 illustrates the graphical solution of Poiseuille Flow at both high (QH = 3 μL/min) and low (QL = 1 μL/min) volume flow rates within the microchannel. As can be seen, the typical parabolic profile of each flow rate is evident, with maximum velocity at the center line. Moreover, the measurements of velocities for individual microbeads are plotted alongside the analytical data to illustrate a less than 7% variance close to the channel walls, but ~15% at the channel center line, as determined by root mean square error. Similarly, the values of imposed shear stress lie along the linear expression defined by the analytical solution for one-dimensional, incompressible flow.

### 3.4. Volume Flow Rates Produced Different Survival Rates for Retinal Endothelial and Retinal Neural Cells

**Figure 7:**
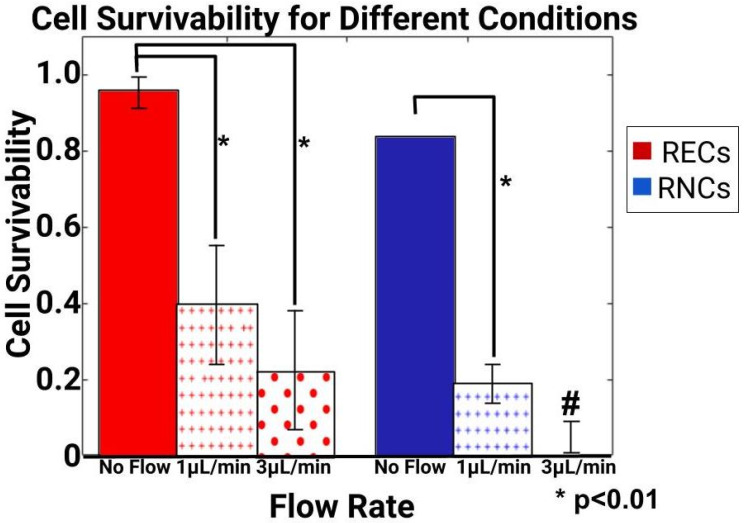
Measurement of cell survival within 3D-printed microdevices after induced flow at low (QL =1 μL/min) and high (QH =3 μL/min) volume flow rates. Retinal endothelial cells (RECs) are shown in red and retinal neural cells (RNCs) are shown in blue. An average of n = 25 measurements were gathered per device, using 5–7 devices per mold. Statistical significance is denoted by *p* < 0.01 (*). # indicates that only trace numbers of cells were observed.

Our tests next seeded retinal endothelial cells (RECs) and retinal neural cells (RNCs) into the interstitial spaces of elastomeric devices fabricated using SLA resin molds. Survival was determined by the Live/Dead assay and DAPI staining for each condition. As seen in Figure 7, RECs exhibited much higher survival rates than RNCs under sustained flow. RECs displayed upwards of 95% survival in control conditions (no flow) but decreased to 40% under low flow conditions of QL = 1 μL/min and 25% when exposed to high volume flow rates of QH = 3 μL/min. Survival at different volume flow rates showed no significant difference between QH and QL but were both significantly different from the control. By contrast, RNCs showed 85% survival in the control, but dropped to 20% under low flow conditions of QL. Furthermore, only trace amounts of DAPI stain was observed in high flow conditions to reflect nearly zero viable cells within the microchannels.

### 3.5. Induced Flow Rates Produced Distinct Cell Morphology Changes

**Figure 8:**
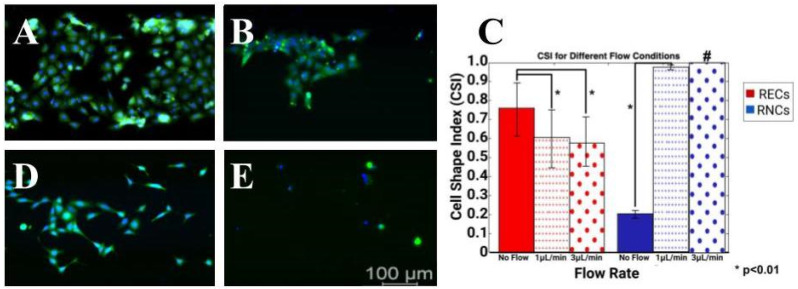
Flow-induced morphology changes of retinal endothelial cells (RECs) and retinal neural cells (RNCs) within 3D-printed microdevices. Representative images of RECs cultured in (**A**) low volume flow rate (QL) and (**B**) high volume flow rate (QH) alongside representative images of RNCs cultured in (**C**) control and (**D**) low volume flow rate (Scale bar = 100 μm). (**E**) Changes in cell morphology measured via cell shape index (CSI) shown after respective induced flow rates. A total of n = 25 cells were used within 3–5 different devices. Statistical significance of * denotes *p* < 0.01. # indicates that only trace numbers of cells were observed.

The final set of experiments applied different volume flow rates of QH and QL upon monolayers of RECs and RNCs seeded within PDMS elastomers produced from SLA resin molds. RNCs were observed to be nearly confluent in control conditions (no flow) but began to display larger surface areas devoid of cells with an increasing flow rate. Interestingly, Figure 8A,B show that while the cell survival rate of RECs decreased with an increased flow rate, the cell morphology became more elongated, as reflected by CSI values that approached 0. Furthermore, changes in CSI values were insignificant between low and high flow rate conditions (*p* > 0.05). By contrast, RNCs were more strongly affected by induced flow. As shown in Figure 8D,E, significant numbers of cells did not survive the low flow rate and only trace amounts of DAPI were recorded to identify viable cells after exposure to high volume flow rates. Moreover, average CSI values of RNCs significantly increased to approach a value of 1 under QL to indicate fully rounded cells typical of apoptosis or detachment.

## 4. Discussion

The broad adaptation of microfluidics in life science applications has facilitated the integration of microscale systems into numerous research platforms. Recent organs-on-chip [82,83,84] and micro-physiological systems [85,86] have elevated microtechnology to critical platforms for the study of human development and adult physiology. Moreover, they have generated excitement for applications of complex bioengineering systems in diverse physiology, including cardiovascular, orthopedics, dental, the nervous system, and more [87,88,89]. Interestingly, rising bio-adaptation of microtechnology has created a growing community at the micro- and mesoscale using biosystems independent of specialized clean room environments and costly equipment. The study of physiological and anatomical structures with characteristic lengths between cells and tissues (approximately 50 μm to 500 μm) are particularly well suited for devices fabricated using lower cost and accessible fabrication techniques, such as 3D printing, to enable fundamental microfluidic cell study across disciplines.

This project is among the first to use physiological flow rates to examine the response of cells in neurovascular barriers of the retina. Despite many excellent systems, few have incorporated applied flow, as per in vivo. This project examined the application of rapid prototyped molds to develop elastomeric systems able to validate these understudied cellular responses. All processing began with a digital model of the desired product, our previously established glial line (gLL) system, and the selection of a material for manufacture. Stereolithography (SLA) and fused deposition modeling (FDM) were chosen for this study (Figure 3) from over a dozen commercial 3D-printing techniques available due to low cost and wide availability for both high and low resource settings, e.g., universities, community colleges, and high schools [90,91]. Molds in this study manufactured via metal milling, SLA, and FDM illustrated similar dimensions for the radius and height of device reservoirs (Table 2), but larger variation in microchannel cross section and hydraulic diameter. Both FDM and SLA have been reported to produce micro- and mesoscale channels effectively, and our study quantitatively measured differences in height-to-width ratio and variance in our devices to precisely verify variations between fabrication methods.

In FDM, the material was melted down and nozzle-extruded to form a two-dimensional layer on the print bed. While nozzle sizes have been traditionally susceptible to blockage, their ability to print smaller features while using less material has made them adaptable to studies on the mesoscale [35], and hence studied here. FDM molds were produced using polyethylene terephthalate (PETG), which is a thermoplastic polyester commonly selected for its durability and formability. The material costs were low, ranging between USD 20–50 per kg, while the rapid prototyping produced plastic molds in short time frames (~2 h). However, the larger height-to-width ratio measured during testing increased the microchannel volume by a third. This result was likely due to the commercial 3D printer used, as this project utilized a machine typical in undergraduate teaching laboratories to examine the capabilities of more cost-effective and widespread printers. However, a 3D-printing system with higher resolution and precision would undoubtedly increase the cost of the devices but produce devices with similar height-to-width ratios, albeit with likely less variation. In addition, the surface roughness of these devices was significantly higher than that of devices manufactured using metal molds or resin molds (Figure 5). The increased roughness was similar for devices functionalized with a laminin coating as a basement membrane for cell adhesion, illustrating lower applicability for cell study overall.

By contrast, PDMS devices produced using resin molds printed via SLA exhibited microchannels with square cross sections. While the dimensions increased the interstitial volumes of the channels, square cross sections are better represented by hydraulic diameter than are rectangular channels, suggesting a higher correlation with flow analyses. In addition, a similar surface roughness to the original metal mold used was found, with and without a laminin coating (Figure 5). As shown, the resin structure is readily visible in a light microscope, but the roughness of its inner channel surfaces approached that of the metal molds. Our study used polylactic acid (PLA), which is a common resin reliable for larger prints, such as splints, implants, dentures, and mouth adaptors [92,93,94] but is also able to produce finer features now used for bioscaffolds [95,96]. The cost of PLA was similar to PETG at USD 40–60 per kg, although more specialized resins can cost upwards of USD 400 per kg. It is noted that a strong limitation of SLA additive manufacturing is the increased curing time (>6 h) required.

Our project then continued by using SLA-produced molds to enable the flow-based study of retinal cells. Flow within the PDMS devices was readily enabled through Luer Locks and conventional tubing connected to a syringe pump (Figure 4). Flow was first measured using microbeads at different volume flow rates to illustrate the measured bulk velocities that were within 7% of one-dimensional analytical flow solutions (Figure 6). We note that the larger cross section of elastomers manufactured using 3D-printed molds versus metal molds may contribute to increased variance with one-dimensional flow approximations, especially with microchannels of shorter lengths. However, the larger size of the device reservoirs introduced an added benefit that may reduce hydrostatic pressure in non- or low-flow systems, as well as improve reagent recycling [6,97].

Next, analytically determined values of shear stresses imposed by those flows were tabulated to continue with the flow-based study of both retinal endothelial cells (RECs) and retinal neural cells (RNCs) significant to the BRB. The viability measurements illustrated that RNCs were more strongly affected by shear stress than RECs (Figure 7). While sustained flow decreased the survival of both cell types, few RNCs were able to survive at a sustained flow at QH, leading to only trace amounts of viable cells. This result is in line with in vivo conditions, where cells reside within retinal tissue and are thereby exposed to minute, interstitial flows much lower than the capillary flow rates upon RECs [69,76]. The results of RECs’ survival were was also of note, as the data showed no difference between the QH and QL flow rates used. This may indicate that the flow rates applied did not approximate in vivo conditions accurately. We note that this is a large limitation of many commercial syringe pumps, which are unable to apply sustained ultra-low flow rates when connected to microchannels via capillary tubing. An interface with a specialized (and more costly) flow apparatus will greatly increase the applicability of this BRB flow study.

The final tests applied external flow rates to show that the cell shape index (CSI) of RECs increased to approach a value of 1, indicative of purely elongated cells (Figure 8). While numerous studies have illustrated the response of endothelial cells to external shear stresses (reviewed in [76]), few studies have considered this behavior in the flow-based study of the BRB. Cell shape becomes critical for the study of RECs’ monolayers on the retinal barrier, as changes in morphology have been correlated with differences in resistivity that may impact BRB integrity and lead to vision loss [73]. Three-dimensionally printed molds can thereby produce cost-effective devices to study different types of shear stress induced via extracellular fluids appropriate to retinal disorders, including fluids with high glucose and those with an accumulation of blood-borne inflammatory factors associated with metabolic disorders, such as diabetes [98].

## 5. Conclusions

Three-dimensionally printed resin molds produced microchannel surfaces able to support the flow-based study of RECs and RNCs. These lower-cost devices enable the fundamental biological study of these underexplored cell groups by researchers without backgrounds in fabrication. Greater adoption of rapid prototyping for the study of physical structures on the micro- and mesoscale will greatly accelerate the development of biomedical therapies across neurovascular barriers.

## Figures and Tables

**Table 1:** Summary of recent microfluidic systems fabricated using varied techniques and applied to the study of retinal cell behaviors.

Author (Year)	Fabrication and Material	Cell Type	Application	Ref
Su et al. (2015)	Photolithography, PDMS	Retinal Neural Cells (RNCs)	Synaptic Guiding	[56]
Mishra et al. (2015)	Photolithography, PDMS	RNCs	Chemotaxis	[57]
Chan et al. (2015) 26024114	Laser Engraving, PMMA	Retinal Ganglion Cells	Drop Delivery	[58]
McCutcheon et al. (2017)	Photolithography, PDMS	RNCs	Adhesion, Migration	[59]
Li et al. (2017) 28612282	Photolithography, PDMS	Endothelial Cells	Microvascular	[60]
Mishra et al. (2017)	Photolithography, PDMS	RNCs	Electrotaxis	[61]
Thakur et al. (2018)	Photolithography, PDMS	RNCs	Adhesion, clustering	[62]
Wu et al. (2019) 31227762	PMMA, Engraving	Retinal Ganglion Cells	Dendritic branching	[63]
Pena et al. (2019)	Metal Milling	Muller Glia	Hypertrophy, migration	[55]
Xue et al. (2021) 34236056	Resin stereolithography	Retinal Stem Cells	micro-millifluidic bioreactor	[64]
Jahagirdar et al. (2022) 35652558	PDMS layers, Punching	RNCs	Cell-Cell interactions	[65]
Sun et al. (2023) 36963105	Resin stereolithography	Retinal Stem Cells	Differentiation	[66]

**Table 2:** Measurements of critical device parameters from elastomeric devices manufactured using 3D molds of metal, resin (PLA), and plastic (PTEG). Dimensions of microchannel length, height, and width are shown with tolerances alongside the height and diameter of device reservoirs. Changes in the average height-to-width ratio and hydraulic diameter of each microchannel are also calculated. Data reflect averages from n = 25 optical microscopy measurements recorded per parameter within 5–7 microdevices produced from each mold.

Mold Type	Device Microchannel					Device Reservoirs	
	Length (cm)	Height (µm)	Width (µm)	Height-Width Ratio	Hydraulic Diameter (µm)	Height (mm)	Diameter (mm)
Metal	1.48 ± 0.05	239.1 ± 6.75	180.7 ± 4.6	1.32, Rect.	205.7 ± 3.07	4.10 ± 0.05	1.00 ± 0.05
Resin (PLA)	1.21 ± 0.05	250.9 ± 32.8	255.2 ± 8.1	0.98, Square	251.9 ± 14.7	6.73 ± 0.05	1.67 ± 0.05
Plastic (PTEG)	1.19 ± 0.05	398.9 ± 10.8	207.8 ± 4.0	1.92, Rect.	273.3 ± 6.0	6.21 ± 0.05	1.66 ± 0.05

## Data Availability

The original contributions presented in the study are included in the article, further inquiries can be directed to the corresponding author.
